# Mechanical and oral antibiotics bowel preparation for elective rectal cancer surgery: A propensity score matching analysis using a nationwide inpatient database in Japan

**DOI:** 10.1002/ags3.12641

**Published:** 2022-11-29

**Authors:** Takuya Oba, Norihiro Sato, Makoto Otani, Keiji Muramatsu, Kiyohide Fushimi, Jun Nagata, Takayuki Torigoe, Kazunori Shibao, Shinya Matsuda, Keiji Hirata

**Affiliations:** ^1^ Department of Surgery 1, School of Medicine University of Occupational and Environmental Health Kitakyushu Japan; ^2^ Occupational Health Data Science Centre University of Occupational and Environmental Health Kitakyushu Japan; ^3^ Department of Preventive Medicine and Community Health, School of Medicine University of Occupational and Environmental Health Kitakyushu Japan; ^4^ Department of Health Informatics and Policy, Graduate School of Medicine Tokyo Medical and Dental University Tokyo Japan

**Keywords:** bowel preparation, diagnosis procedure combination, low anterior resection, nationwide database, rectal cancer surgery

## Abstract

**Aim:**

The best bowel preparation method for rectal surgery remains controversial. In this study we compared the efficacy and safety of mechanical bowel preparation (MBP) alone and MOABP (MBP combined with oral antibiotic bowel preparation [OABP]) for rectal cancer surgery.

**Methods:**

In this retrospective study we analyzed data from the Japanese Diagnosis Procedure Combination (DPC) database on 37 291 patients who had undergone low anterior resection for rectal cancer from 2014 to 2017. Propensity score matching analysis was used to compare postoperative outcomes between MBP alone and MOABP.

**Results:**

A total of 37 291 patients were divided into four groups: MBP alone: 77.7%, no bowel preparation (NBP): 16.9%, MOABP: 4.7%, and OABP alone: 0.7%. In propensity score matching analysis with 1756 pairs, anastomotic leakage (4.84% vs 7.86%, *P* < 0.001), small bowel obstruction (1.54% vs 3.08%, *P* = 0.002) and reoperation (3.76% vs 5.98%, *P* = 0.002) were less in the MOABP group than in the MBP group. The mean duration of postoperative antibiotics medication was shorter in the MOABP group (5.2 d vs 7.5 d, *P* < 0.001) than in the MBP group. There was no significant difference between the two groups in the incidence of *Clostridium difficile* (CD) colitis (0.40% vs 0.68%, *P* = 0.250) and methicillin‐resistant *Staphylococcus aureus* (MRSA) colitis (0.11% vs 0.17%, *P* = 0.654). There was no significant difference in in‐hospital mortality between the two groups (0.00% vs 0.11% respectively, *P* = 0.157).

**Conclusion:**

MOABP for rectal surgery is associated with a decreased incidence of postoperative complications without increasing the incidence of CD colitis and MRSA colitis.

## Graphical abstract

Using the Japanese National Database, we showed that mechanical and oral antibiotics bowel preparation for rectal cancer surgery is associated with a decreased incidence of postoperative complications without increasing the incidence of CD colitis or MRSA colitis. In fact, mechanical bowel preparation alone is still the most major bowel preparation for rectal cancer surgery in the real world.

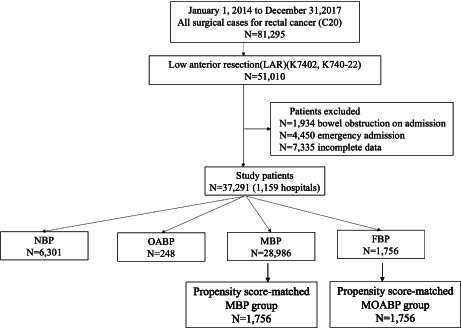

## 1 INTRODUCTION

The use of mechanical bowel preparation (MBP) in combination with oral antibiotic bowel preparation (OABP) before colorectal surgery has been discussed since the 1970 s.^1^ Recently, guidelines based on systematic reviews and reviews on bowel preparation for colorectal surgery have been published around the world.^2^, ^3^, ^4^, ^5^ Those guidelines recommended MBP with OABP (MOABP) based on several reviews showing that MBP alone did not prevent postoperative complications and may be harmful,^6^, ^7^, ^8^, ^9^ while MOABP reduced postoperative complications after colorectal surgery.^10^, ^11^ Evidence of bowel preparation for colorectal surgery is increasing, but there are still few studies focused on rectal surgery. Actually, the Enhanced Recovery After Surgery (ERAS) Society indicated that MBP alone should not be used routinely in colonic surgery, but may be used for rectal surgery.^4^ The risk of anastomotic leakage after rectal surgery is higher than that after colon surgery, and the method of anastomosis is different between colon and rectal surgery. More evidence is needed about appropriate bowel preparation for rectal surgery.

In addition, many surgeons have chosen MBP rather than MOABP in spite of recommendations by the guidelines. One of the reasons is that surgeons concern about enteritis caused by OABP such as *Clostridium difficile* (CD) colitis and methicillin‐resistant *Staphylococcus aureus* (MRSA) colitis.^12^, ^13^ On the other hand, it has been reported that the incidence of CD colitis and MRSA colitis did not increase if oral antibacterial agents are used properly.^14^, ^15^

There are no studies that investigated the bowel preparation for rectal surgery using a nationwide database. We need to research which bowel preparations for rectal surgery are performed in a real‐world and which bowel preparations are useful and safe. In this study we evaluated the current status of bowel preparation for rectal cancer surgery and the efficacy and safety of MOABP compared with MBP in a real‐world setting using the Japanese Diagnosis Procedure Combination (DPC) database.

## 2 MATERIALS AND METHODS

### 2.1 Data source

In this nationwide retrospective study, we used the DPC database from January 2014 to December 2017. It contains discharge abstracts and administrative reimbursement claim data from inpatient cases collected at participating hospitals, and it has been used in various studies^16^, ^17^ The data were collected by the DPC Research Institute (a nonprofit organization) in collaboration with the Ministry of Health, Labor, and Welfare of Japan. The database includes the following data: disease names, hospitalization costs, comorbidities at admission and during hospitalization, coded according to the International Statistical Classification of Diseases and Related Health Problems, 10th revision (ICD‐10), age, sex, length of hospital stay, medical procedures including surgery, names and quantities of medicines administered, and discharge status (including in‐hospital deaths).^18^ Medical procedures are indexed with a Japanese code (K‐code),^19^ assigned by the Ministry of Health, Labor, and Welfare of Japan. This study was approved by the Ethics Committee for Medical Care and Research at the University of Occupational and Environmental Health Japan (R02‐007).

### 2.2 Patient selection

**FIGURE 1:**
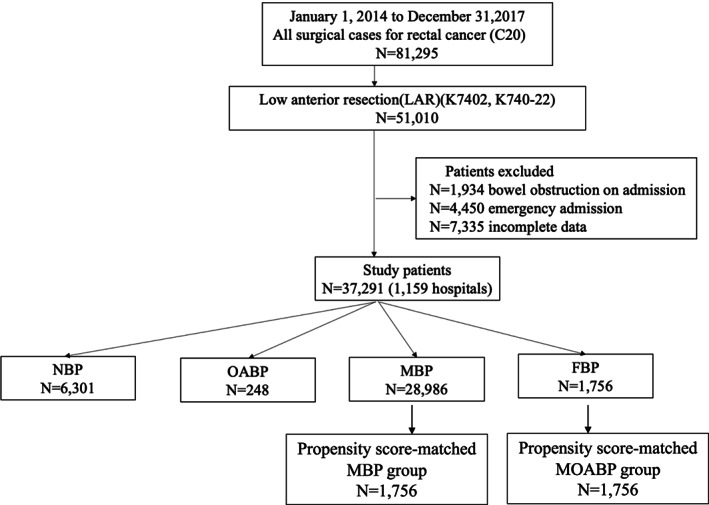
Flow diagram. Between 2014 and 2017, 81 295 patients who underwent rectal cancer surgery were registered in the DPC. In total, 51 010 patients underwent low anterior resection. Patients with bowel obstruction on admission (n = 1934), emergency admission (n = 7335), and without sufficient data (n = 7335) were excluded, leaving 37 291 patients in the study: 6301 no bowel preparation (NBP), 248 oral antibiotics bowel preparation only (OABP), 28 986 mechanical bowel preparation only (MBP), and 1756 patients who received mechanical and oral antibiotics preparation (MOABP). After propensity score matching analysis, 1756 pairs were matched

Inclusion criteria were as follows: inpatient status and admission for rectal cancer (ICD‐10 code: C20), and underwent low anterior resection (LAR) (K‐code: K7402, K740‐22). Exclusion criteria were bowel obstruction on admission, emergency admission and incomplete data. Patients who were given polyethylene glycol (PEG) or magnesium citrate before LAR comprised the MBP group. Patients who were given metronidazole and kanamycin before LAR comprised the OABP group. Patients who were given neither MBP nor OABP comprised the no bowel preparation (NBP) group. Patients who were given both MBP and OABP comprised the MOABP group (Figure 1).

### 2.3 Endpoints

Endpoints were as follows: in‐hospital mortality, 30‐d postoperative mortality, necessity of reoperation with general anesthesia, rate of surgical wound dehiscence, rate of anastomotic leakage, rate of postoperative small bowel obstruction needing a trans‐nasal long decompression tube, necessity of perioperative transfusion, rate of CD colitis, rate of MRSA colitis, length of hospital stay (LOS), duration of postoperative antibiotic administration and total hospitalization cost.

### 2.4 Statistical analysis

We conducted propensity score‐matched (PSM) analysis to compare the efficacy of MOABP with that of MBP. We used the following variables for PSM: sex, age, body mass index (BMI), clinical TNM stage, rate of diverting stoma, rate of laparoscopic surgery, smoking, the use of antidiabetic drugs, oral corticosteroid drugs, oral antiplatelet drugs, and oral anticoagulant drugs in the admission and hospital volume. The hospital volume, which was used as a hospital‐level factor, was defined as the annual number of LAR executed at each facility and categorized into quartiles (low volume: <54 cases, medium volume: 55–95 cases, high volume: 96–147cases, and very high volume: 148–581 cases per 4 y).

Nearest‐neighbor matching was employed with a caliper width equal to 0.2 of the standard deviation of the logit of propensity scores. We subsequently compared in‐hospital mortality, 30‐d postoperative mortality, necessity of reoperation with general anesthesia, rate of surgical wound dehiscence, rate of anastomotic leakage, rate of postoperative small bowel obstruction needing trans‐nasal long decompression tube, necessity of perioperative transfusion, rate of CD colitis, rate of MRSA colitis. Using chi‐square tests, and LOS, duration of postoperative antibiotic administration and total hospitalization cost, using the Mann–Whitney *U*‐test.

Stata Version 15.0 (Stata Corp, College Station, TX, USA) was used for all statistical analyses. A *P* < 0.05 was considered statistically significant.

## 3 RESULTS

### 3.1 The current status of bowel preparation

**FIGURE 2:**
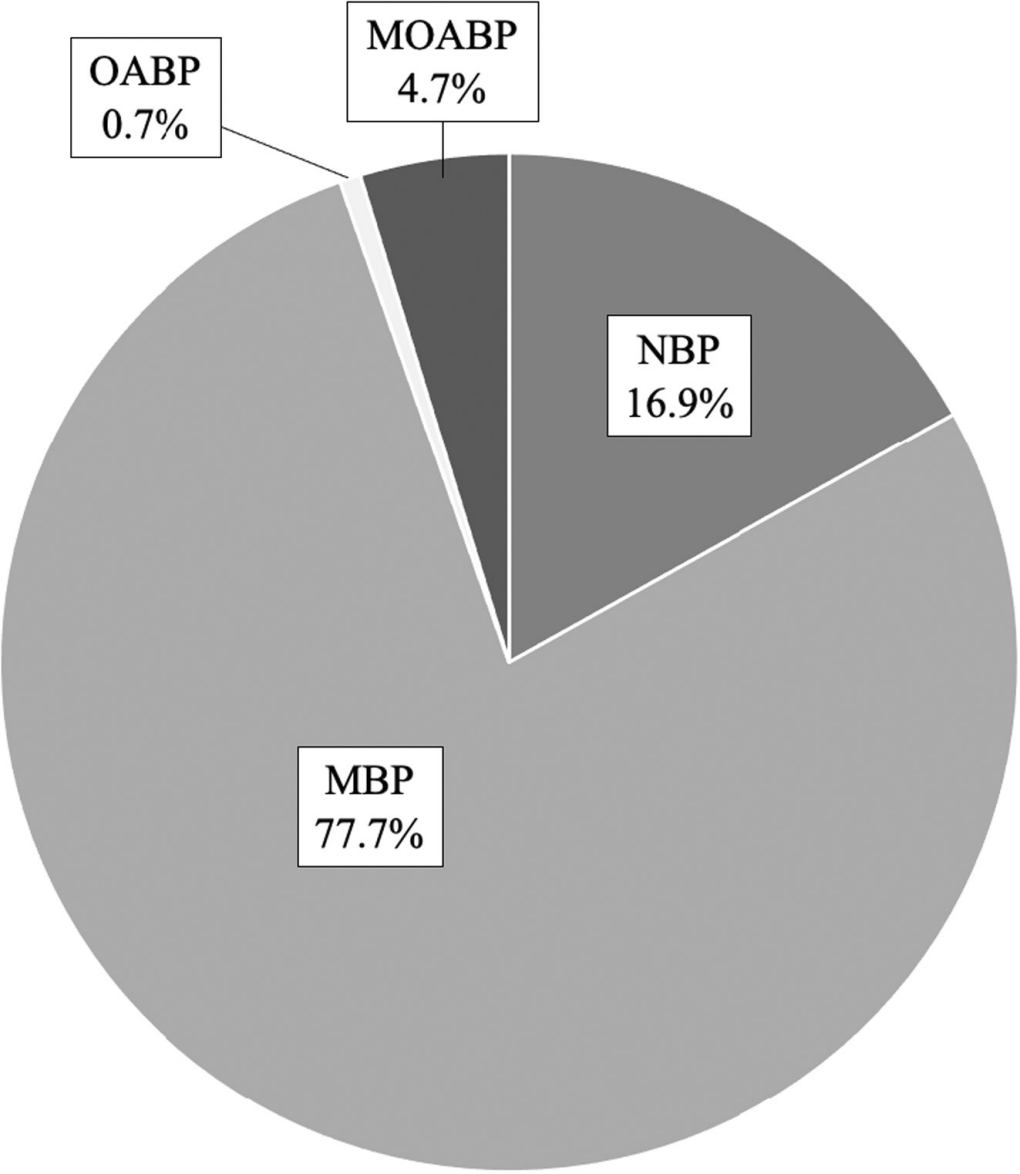
Age distribution of each group. The MBP group accounted for 77.7%. The other groups were as follows: the NBP group 16.9%, the OABP group 0.7% and the MOABP group 4.7%

During the study period, 51 010 patients who underwent low anterior resection for rectal cancer were registered in the DPC database. Patients who had bowel obstruction on admission (1934 patients), or who admitted emergently (4450 patients), or who lacked sufficient data (7335 patients) were excluded, resulting in inclusion of 37 291 patients (1159 hospitals). In all, 37 291 patients were divided into four groups (NBP: 6301 patients, OABP: 248 patients, MBP: 28986 patients, MOABP: 1756 patients) (Figure 1). The ratio of the four groups is shown in Figure 2. The MBP group accounted for 77.7%. The other groups were as follows: The NBP group 16.9%, the OABP group 0.7%, and the MOABP group 4.7%.

### 3.2 Patient characteristics

Patient characteristics of each group are shown in Table 1. The NBP group tended to include advanced clinical TNM stage (Stages III or IV) patients and patients who underwent LAR at very high‐volume hospitals. The MOABP group tended to be higher for the laparoscopic surgery rate than other groups and included patients who underwent LAR at high or very high‐volume institutions.

**TABLE 1:** Baseline characteristics according to the type of bowel preparation

	NBP (n = 6301)		MBP (n = 28 986)		MOABP (n = 1756)	
	n	%	n	%	n	%
Sex, male	4219	66.96	77.57	66.01	1152	65.60
Age, y, mean (SD)	66.6	(10.9)	66.8	(10.9)	66.5	(11.0)
BMI, kg/m^2^						
<18.5	532	8.44	2463	8.50	154	8.77
18.5–25	4624	73.39	20 074	69.25	1203	68.51
>25	1145	18.17	6449	22.25	399	22.72
Clinical TNM Stage						
I	1806	28.66	10 428	35.98	652	37.13
II	1497	23.76	6854	23.65	437	24.89
III	2369	37.60	9466	32.66	540	30.75
IV	629	9.98	2238	7.72	127	7.23
Diverting stoma	1809	28.71	7182	24.78	537	30.58
Laparoscopic	4767	75.65	22 603	77.98	1524	86.79
Smoker	3292	52.25	15 078	52.02	944	53.76
Antidiabetic drugs	1067	16.93	5686	19.62	363	20.67
Oral corticosteroid drugs	100	1.59	446	1.54	26	1.48
Oral antiplatelet drugs	391	6.21	2164	7.47	145	8.26
Oral anticoagulant drugs	214	3.40	1174	4.05	80	4.56
Hospital volume						
Low	1448	22.98	7483	25.82	311	17.71
Middle	1622	25.74	7107	24.52	327	18.62
High	1067	16.93	7477	25.80	615	35.02
Very high	2164	34.34	6919	23.87	503	28.64

Abbreviations: MBP, mechanical bowel preparation; MOABP, mechanical bowel preparation with oral antibiotic bowel preparation; NBP, no bowel preparation.

### 3.3 Postoperative outcomes before propensity score matching

Table 2 shows the postoperative outcomes in each group. In‐hospital mortality (0.48%), 30‐d postoperative mortality (0.33%), necessity of reoperation with general anesthesia (6.89%), rate of surgical wound dehiscence (2.16%), rate of anastomotic leakage (9.67%), and necessity of perioperative transfusion (7.28%) were the highest in the NBP group among the groups. The mean of LOS (24.1 d) was the longest in the NBP group. On the other hand, in‐hospital mortality (0.00%), 30‐d postoperative mortality (0.00%), necessity of reoperation with general anesthesia (3.76%), rate of surgical wound dehiscence (0.85%), rate of anastomotic leakage, (4.84%), rate of postoperative small bowel obstruction needing a trans‐nasal long decompression tube (1.54%), and necessity of perioperative transfusion (5.81%) were the lowest in the MOABP group among the groups. In addition, the mean of LOS (22.3 d) was the shortest in the MOABP group.

**TABLE 2:** Postoperative outcomes according to type of bowel preparation

	NBP (n = 6301)		MBP (n = 28 986)		MOABP (n = 1756)	
Postoperative outcomes	n	%	n	%	n	%
Surgical wound dehiscence	136	2.16	535	1.85	15	0.85
Anastomotic leakage	609	9.67	2316	7.99	85	4.84
Small bowel obstruction	165	2.62	820	2.83	27	1.54
Transfusion	459	7.28	2029	7.00	102	5.81
CD colitis	25	0.40	133	0.46	7	0.40
MRSA colitis	11	0.17	24	0.08	2	0.11
Reoperation with general anesthesia	434	6.89	1759	6.07	66	3.76
30‐d mortality	21	0.33	31	0.11	0	0
In‐hospital mortality	30	0.48	68	0.23	0	0
LOS (d)	24.1^a^	20.0^b^	23.8^a^	18.1^b^	22.3^a^	13.0^b^
Duration of postoperative antibiotics medication (d)	7.3^a^	13.4^b^	7.3^a^	14.6^b^	5.2^a^	10.3^b^
Total hospitalization cost (yen)	1811027^a^	870809^b^	1974936^a^	697535^b^	1923007^a^	598316^b^

Abbreviations: CD, *Clostridium difficile*; LOS, length of hospital stay; MBP, mechanical bowel preparation; MOABP, mechanical bowel preparation with oral antibiotic bowel preparation; MRSA, methicillin‐resistant *Staphylococcus aureus*; NBP, no bowel preparation.

^a^ Mean.

^b^ SD.

### 3.4 Comparison of postoperative outcomes between MOABP and MBP after propensity score matching

The patient characteristics after PSM are summarized in Table 3. A total of 1756 pairs were matched (Figure 1). We confirmed that the characteristics of both groups were similar after PSM. The postoperative outcomes after PSM are shown in Table 4. The rate of anastomotic leakage was significantly lower in the MOABP group than in the MBP group (4.84% vs 7.86%, *P* < 0.001). Also, the rate of postoperative small bowel obstruction needing a trans‐nasal long decompression tube (1.54% vs 3.08%, *P* = 0.002) and the necessity of reoperation with general anesthesia (3.76% vs 5.98%, *P* = 0.002) were significantly lower in the MOABP group than in the MBP group. The duration of postoperative antibiotics medication was significantly shorter in the MOABP group than in the MBP group (mean 5.2 d vs 7.5 d, *P* < 0.001). No significant differences were found between the MOABP and the MBP group in terms of the rate of CD colitis (0.40% vs 0.68%, *P* = 0.250) and the rate of MRSA colitis (0.11% vs 0.17%, *P* = 0.654). There were no significant differences in the in‐hospital mortality, 30‐d postoperative mortality, the rate of surgical wound dehiscence, necessity of perioperative transfusion, LOS, and total hospitalization cost between the MOABP groups and the MBP groups.

**TABLE 3:** Characteristics after propensity score matching

	Propensity‐matched patients (n = 3512)				*P*
	MBP (n = 1756)		MOABP (n = 1756)		
	n	%	n	%	
Sex, male	1150	65.49	1152	65.60	0.943
Age, y, mean (SD)	66.3	(11.0)	66.6	(10.9)	0.799
BMI, kg/m^2^					
<18.5	149	8.49	154	8.77	0.825
18.5–25	1220	69.48	1203	68.51	
>25	387	22.04	399	22.72	
Clinical TNM Stage					
I	663	37.76	652	37.13	0.628
II	409	23.29	437	24.89	
III	564	32.12	540	30.75	
IV	120	6.83	127	7.23	
Diverting stoma	551	31.38	537	30.58	0.609
Laparoscopic	1525	86.85	1524	86.79	0.960
Smoker	933	53.13	944	53.76	0.710
Antidiabetic drugs	361	20.56	363	20.67	0.934
Oral corticosteroid drugs	29	1.65	26	1.48	0.683
Oral antiplatelet drugs	137	7.80	145	8.26	0.619
Oral anticoagulant drugs	75	4.27	80	4.56	0.681
Hospital volume					
Low	307	17.48	311	17.71	0.106
Middle	367	20.90	327	18.62	
High	554	31.55	615	35.02	
Very high	528	30.07	503	28.64	

Abbreviations: MBP, mechanical bowel preparation; MOABP, mechanical bowel preparation with oral antibiotic bowel preparation.

**TABLE 4:** Postoperative outcomes after propensity score matching

	MBP (n = 1756)		MOABP (n = 1756)		*P*
Postoperative outcomes	n	%	n	%	
Surgical wound dehiscence	27	1.54	15	0.85	0.062
Anastomotic leakage	138	7.86	85	4.84	<0.001
Small bowel obstruction	54	3.08	27	1.54	0.002
Transfusion	111	6.32	102	5.81	0.525
CD colitis	12	0.68	7	0.40	0.250
MRSA colitis	3	0.17	2	0.11	0.654
Reoperation with general anesthesia	105	5.98	66	3.76	0.002
30‐d mortality	1	0.06	0	0.00	0.317
In‐hospital mortality	2	0.11	0	0.00	0.157
LOS (d)	23.6^a^	22.4^b^	22.3^a^	13.0^b^	0.605
Duration of postoperative antibiotics medication (d)	7.5^a^	19.4^b^	5.2^a^	10.3^b^	<0.001
Total hospitalization cost (yen)	1984608^a^	856076^b^	1923007^a^	598316^b^	0.307

Abbreviations: CD, *Clostridium difficile*; LOS, length of hospital stay; MBP, mechanical bowel preparation; MOABP, mechanical bowel preparation with oral antibiotic bowel preparation; MRSA, methicillin‐resistant *Staphylococcus aureus*.

^a^ Mean.

^b^ SD.

## 4 DISCUSSION

In the present study we investigated the current status of bowel preparation and the effect of MOABP for elective rectal cancer surgery in a real‐world setting using a large, nationwide administrative database. To our knowledge, this study is one of the largest observational studies including only patients undergoing rectal cancer surgery.

The major findings were as follows: (a) In Japan, MBP is still the major bowel preparation for rectal surgery. (b) MOABP results in a significantly lower incidence of anastomotic leakage, postoperative small bowel obstruction, and reoperation than MBP. (c) MOABP does not affect CD colitis and MRSA colitis.

In this study we found that the MBP group was the majority and accounted for 77.7% among all bowel preparation types. On the other hand, the MOABP group accounted for only 4.7%. Regarding the patient characteristics between the MBP group and the MOABP group, there was a notable difference in the rate of patients who underwent LAR at the high or very high‐volume hospitals. We found that surgeons at high or very high‐volume hospitals tended to choose MOABP more frequently. The high rate of laparoscopic surgery in the MOABP group is thought to be affected by hospital volumes. The NBP group accounted for 16.9%. The NBP group included more cases of advanced cancer. It is expected that the NBP group included many patients who could not receive MBP because of the possibility of intestinal obstruction caused by MBP. Along with that, the postoperative outcomes of the NBP group also seem to be worse than the MBP group and the MOABP group. We could not match the NBP group and other groups statistically because there were many factors that could not be adjusted. Furthermore, as we were concerned that patients of the NBP group had many factors that affect postoperative outcomes, we did not include this group for further comparison.

Even after matching factors of patients and hospitals with PSM, the MOABP group was shown to be superior to the MBP group in terms of anastomotic leakage, postoperative small bowel obstruction and reoperation, and the duration of postoperative antibiotics medication. Some studies showed that the use of MOABP reduced the incidence of surgical site infection (SSI), anastomotic leakage, small bowel obstruction, and hospital readmission before elective rectal surgery compared with the use of MBP.^11^, ^20^, ^21^ Kiran et al presented that the effect of antibiotics on leak rate may be explained by fewer clinically evident events as opposed to actual leaks due to reduced intraabdominal bacterial burden and less subsequent contamination after leakage.^11^ In the current study, we revealed that MOABP significantly reduced anastomotic leakage, postoperative small bowel obstruction, and reoperation in rectal surgery, consistent with the results of previous studies.

In addition, MOABP did not increase CD colitis and MRSA colitis compared with MBP. The OABP component has been cited to increase the risk of CD colitis and MRSA colitis following surgery.^22^, ^23^, ^24^ However, there are studies that OABP do not enhance postoperative enterocolitis if appropriate administration is used for a short period of time,^25^ and studies that use of metronidazole rather suppresses CD colitis.^26^ The results of this study also show that the safety of MOABP is not inferior to that of MBP.

This study has several limitations that had been reported in the previous studies using the DPC database.^27^, ^28^, ^29^ First, this study was an observational and retrospective study. Hoshino et al presented that case‐matched studies and cohort studies have a potential to overestimate the treatment effect, compared with a randomized clinical trial (RCT).^30^ However, we analyzed an extra‐large number of patients, so this limitation could be complemented. Second, we could not directly measure SSI. Surgical wound dehiscence corresponds with deep incisional SSI. We could not research superficial incisional SSI. Third, it is unlikely that both MOABP and MBP alone were performed in the same institution. The differences between institutions affect the postoperative results. Finally, there is a lack of detailed information regarding the bowel preparation. For example, the duration of preparation and the dose are not included.

In conclusion, our investigation using real‐world data highlight the effectiveness of MOABP for elective rectal cancer surgery. MOABP decreased the incidence of anastomotic leakage, postoperative small bowel obstruction, and reoperation without increasing the incidence of CD colitis and MRSA colitis.

## DISCLOSURES

Funding Information: This study was supported by research funding from the Department of Surgery 1 at the School of Medicine, University of Occupational and Environmental Health.

Conflict of Interest: The authors have no conflicts of interest to disclose.

Ethical Approval: This study was approved by the Ethics Committee for Medical Care and Research at the University of Occupational and Environmental Health Japan (R1‐067).
